# Gut microbiota alteration was related to subclinical hypothyroidism and dyslipidemia in mice

**DOI:** 10.1096/fj.202402289RR

**Published:** 2025-03-07

**Authors:** Ru Wang, Xiaqing Yu, Haidong Cai, Ganghua Lu, Dingwei Gao, Mengyu Zhang, Li Chai, Wanwan Yi, Zhongwei Lv

**Affiliations:** ^1^ Clinical Nuclear Medicine Center, Imaging Clinical Medical Center, Institute of Nuclear Medicine, Institute of Clinical Mass Spectrometry Applied Research Center, Department of Nuclear Medicine, Shanghai Tenth People's Hospital, School of Medicine Tongji University Shanghai China; ^2^ Shanghai Public Health Clinical Center Fudan University Shanghai China

**Keywords:** 16S rRNA gene sequencing, dyslipidemia, gut microbiota, subclinical hypothyroidism, thyroid function

## Abstract

Gut microbiota has a close connection to different thyroid disorders, yet research on its links to subclinical hypothyroidism (SCH) remains limited and insufficient. In this study, we explored the potential relationship between the gut microbiota and SCH, as well as dyslipidemia in SCH mice. The SCH mouse model was induced using methimazole. The composition of the gut microbiota from mice was then analyzed through 16S rRNA gene sequencing technology. An antibiotic disruption experiment was used to assess how gut microbiota imbalance impacts thyroid function. The SCH mouse models were constructed and accompanied by significant dyslipidemia. The results revealed no significant differences in the *Firmicutes* to *Bacteroidota* ratio or α‐diversity in gut microbiota from SCH and control mice, and in β‐diversity, there was a noticeable but small difference between the groups. 14 differential genera between the two groups identified through LEfSe analysis were significantly correlated with serum lipid levels. Furthermore, the results of the antibiotic disruption experiment demonstrated that gut microbiota imbalance exacerbated the hypothyroidism in mice. The present results suggest that subclinical hypothyroidism has not yet caused significant changes in gut microbiota homeostasis, but gut microbiota plays an important role in regulating thyroid function and is closely associated with dyslipidemia in SCH. This study could help understand the relationship between gut microbiota and SCH, and offer new perspectives on dyslipidemia management in SCH.

## Graphical abstract

This study showed no significant changes in gut microbiota homeostasis in subclinical hypothyroidism (SCH) mice induced by methimazole. However, the differential genera between the control and SCH mice were closely linked to elevated serum lipid levels in SCH. Furthermore, gut microbiota imbalance exacerbated hypothyroidism, suggesting that gut microbiota plays an important role in regulating thyroid function. This study could help us understand the relationship between gut microbiota and SCH, and offer new perspectives on dyslipidemia management in SCH.

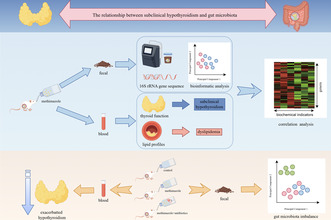

## 1 INTRODUCTION

Subclinical hypothyroidism (SCH) is a common, mild thyroid dysfunction disorder, characterized by raised serum thyroid stimulating hormone (TSH) levels, while serum free thyroxine (FT4) levels remain normal. The prevalence of SCH varies widely across studies, from 4% to 20%, and is more common in females, older individuals, and those in iodine‐deficient regions.^1^, ^2^, ^3^, ^4^ Patients with SCH manifest insidious clinical symptoms and are biochemically diagnosed solely based on thyroid function tests. Furthermore, the process of subclinical hypothyroidism is often accompanied by dyslipidemia, such as increasing levels of total cholesterol (TC), triglycerides (TG), and low‐density lipoprotein cholesterol (LDL‐C).^5^, ^6^, ^7^ Previous research has demonstrated that SCH elevates the risk of cardiovascular diseases and serves as an independent risk factor for atherosclerosis and myocardial infarction in elderly women.^8^, ^9^ Therefore, it is crucial to monitor and address the progression of subclinical hypothyroidism.

Numerous studies have revealed that gut microbiota has a relationship with various diseases in human.^10^, ^11^, ^12^ Thyroid hormone can target the gut, and gut microbiota is essential for peripheral thyroid hormone metabolism.^13^, ^14^ Previous research has shown that gut microbiota is associated with a variety of thyroid diseases, such as Graves' disease, Hashimoto's thyroiditis, and thyroid cancer, among others, and has put forward the concept of the “gut‐thyroid axis.”^15^, ^16^, ^17^ Nevertheless, studies investigating gut microbiota in SCH remain scarce and are predominantly observational research in the diseased state.^18^, ^19^, ^20^ For example, Yao et al. found that the L‐thyroxine dose required to maintain stable TSH levels in SCH patients might be associated with the composition of gut microbiota.^18^ Interestingly, an animal study involving surgery‐induced hypothyroidism (SHO) rats found that the gut microbiota of the SHO group showed clear clustering, distinctly separate from the sham operation (SHAM) group at week 4 after thyroidectomy (overt hypothyroidism), but returned to a dispersed pattern, closely aligning with the SHAM group at week 7 after thyroidectomy (subclinical hypothyroidism).^20^ Therefore, the relationship between gut microbiota and SCH, a mild form of thyroid dysfunction, especially the role of gut microbiota in SCH, merits further investigation to advance the understanding of the “gut‐thyroid axis.”

Additionally, many studies have demonstrated that thyroid hormone and TSH are important regulatory factors in the regulation of lipid metabolism.^6^ At present, the relevant mechanism studies for dyslipidemia in hypothyroidism mainly focus on the related pathways of thyroid hormone and TSH. Notably, a larger number of studies have shown that gut microbiota is closely associated with hyperlipidemia and is essential for regulating lipid metabolism.^21^, ^22^ Moreno‐Indias et al. found that patients with metabolic syndrome, which was characterized by hyperlipidemia, had higher levels of LPS‐producing bacteria (like *Escherichia coli* and *Enterobacter cloacae*) and lower levels of potential probiotics (such as *Faecalibacterium prausnitzii*, *Bifidobacterium*, and *Lactobacillus*) in their feces compared with healthy individuals.^23^ Gargari et al. observed that alterations in lipid levels were associated with changes in certain bacterial groups, including *Lachnospiraceae*, *Ruminococcaceae*, *Akkermansia*, *Bacteroides*, *Roseburia*, and *Faecalibacterium*, in children and adolescents with primary hyperlipidemia.^24^ Studies using probiotics or fecal microbiota transplantation (FMT) provide compelling evidence for the involvement of gut microbiota in regulating lipid metabolism. A study of 70 healthy adults found that daily *Lactobacillus plantarum* Q180 supplements could significantly improve post‐meal blood lipid levels (including LDL‐C and TC) by altering the composition of gut microbiota.^25^ Similarly, another clinical trial revealed that the combination of three *Lactobacillus plantarum* strains effectively reduced TC levels in patients.^26^ A recent animal study demonstrated that the FMT from Paeoniflorin‐treated hyperlipidemic mice improved lipid metabolism disorder and mitigated liver lipid accumulation and damage in hyperlipidemic mice.^27^ These studies hint that gut microbiota may be involved in the alteration of serum lipid levels in SCH individuals, which has not been verified.

Therefore, in the present study, we utilized a chemically induced SCH mouse model combined with antibiotic disruption to study the connection between the intestinal flora and subclinical hypothyroidism as well as dyslipidemia in SCH.

## 2 MATERIALS AND METHODS

### 2.1 Animal

Female BALB/c mice aged six to eight weeks were purchased and raised in Shanghai Tenth People's Hospital's Animal Experiment Center. All animal were raised in a temperature and humidity‐controlled laminar flow room under a 12‐h light/dark cycle, and they were allowed to eat and drink as they pleased. In order to construct the SCH mouse model, drinking water was treated with methimazole (MMI, 75 mg/L) for 12 weeks. A combination of MMI (75 mg/L), polymyxin B (75 mg/L), and neomycin (150 mg/L) was continuously added to the drinking water for 12 weeks in the antibiotic disruption experiment. Shanghai Tenth People Hospital's Animal Ethics Committee approved these experiments according to the Guidelines for the Care and Use of Laboratory Animals (approval no. SHDYY‐2021‐2801).

### 2.2 Sample collection

Following 12 weeks of experimental intervention, the fecal sample of each mouse was collected individually and promptly kept at −80°C. Mice were anesthetized after fasting for 6 h. Subsequently, blood samples were obtained by removing the eyeballs of anesthetized mice, followed by swift cervical dislocation to euthanize the mice. To isolate serum, collected whole blood was centrifuged for 10 min at 4°C at 3000 rpm after being clotted at room temperature for 1 h and finally stored at −80°C to be analyzed further.

### 2.3 Serum biochemical analysis

Serum levels of TSH and FT4 were quantified using mouse TSH and mouse FT4 ELISA kits, respectively, as per the manufacturer's protocols. Additionally, liver function parameters (including alanine aminotransferase (ALT) and aspartate aminotransferase (AST)) and lipid profiles (TC, TG, LDL‐C, and high‐density lipoprotein cholesterol (HDL‐C)) were measured in mouse serum using the corresponding assay kits as directed by the manufacturer.

### 2.4 16S ribosomal RNA (rRNA) gene sequencing

TGuide S96 magnetic bead soil/fecal genomic DNA extraction kit was used to extract the DNA in the fecal samples, and DNA concentration was assessed by a microplate reader. Subsequently, PCR was used to carry out the full‐length 16S rRNA gene using the primer 16S_27F (5′‐AGRGTTTGATYNTGGCTCAG‐3′) and 16S_1492R (5′‐TASGGHTACCTTGTTASGACTT‐3′). The PCR reactions employed KOD‐FX‐Neo DNA polymerase. The PacBio SMRTbell Template Prep Kit (PacBio, USA) was used to construct a sequencing library after the purification, quantification, and homogenization of PCR products. Finally, at Shanghai Baiqu Biomedical Technology Co., LTD, the PCR products derived from all fecal samples were sequenced on the PacBio Sequel II platform.

### 2.5 Bioinformatic analysis of sequencing data

The original sequencing data were identified by barcode sequence using lima (v 1.7.0) software, and underwent length‐based filtering using cutadapt (version 1.9.1) and the elimination of chimeric sequences using UCHIME (v 4.2) software, resulting in high‐quality sequencing data suitable for subsequent analysis. By computing 97% similarity between sequences, USEARCH software (v 10.0) demultiplexed and clustered them into operational taxonomic units (OTUs). Taxonomic information of OTUs were conducted using the SILVA database (release138). A software package called Quantitative Insights into Microbial Ecology (QIIME 2) (https://qiime2.org/) was used to assess both alpha (α) diversity and beta (β) diversity. Based on the weighted and unweighted algorithms, β‐diversity analysis was visualized using non‐metric multidimensional scaling (NMDS) or principal coordinate analysis (PCoA). The unweighted algorithms (binary Jaccard distance and unweighted Unifrac distance) consider species presence/absence, while the weighted algorithms (Bray‐Curtis distance and weighted Unifrac distance) account for species abundance alongside presence/absence. Furthermore, this study used linear discriminant analysis (LDA) effect size (LefSe) to identify taxa exhibiting dissimilar relative abundances among groups.

### 2.6 Statistical analysis

Unless specified, data are presented as means ± standard error of the mean (SEM). The Shapiro–Wilk normality test was used to test the normality of data. Statistical significance between the two groups was assessed using the independent sample t‐test for normal data (TSH, FT4, AST, TG, LDL‐C, HDL‐C, Chao1 index, Ace index, and Shannon index) and the Mann–Whitney U test for non‐normal data (ALT, TC, and Simpson index). ANOVA with one‐way differences was used for multiple group comparisons, followed by the LSD test for post hoc comparisons. For the correlation analysis, correlations between the relative abundance of genera and serum biochemical indexes were conducted using Spearman correlation analysis; the results were visualized as a heatmap using the corrplot package (v.0.84) in R software (v.3.6.3). Correlations between α‐diversity indices (Chao1 index, Ace index, Shannon index, Simpson index) and serum biochemical indices (TSH, FT4, TC, TG, LDL‐C, HDL‐C) were analyzed using Pearson correlation analysis, except for the correlation between TC and α‐diversity indices or between the Simpson index and serum biochemical indices, which used Spearman correlation analysis. It was assumed that a statistically significant difference could be determined if the *p*‐value is less than .05. Unless otherwise specified, analyses were conducted with IBM SPSS 26.0 for Windows (IBM Corp, Armonk, NY, USA).

## 3 RESULTS

### 3.1 Serum biochemical indices in mice with subclinical hypothyroidism

**FIGURE 1:**
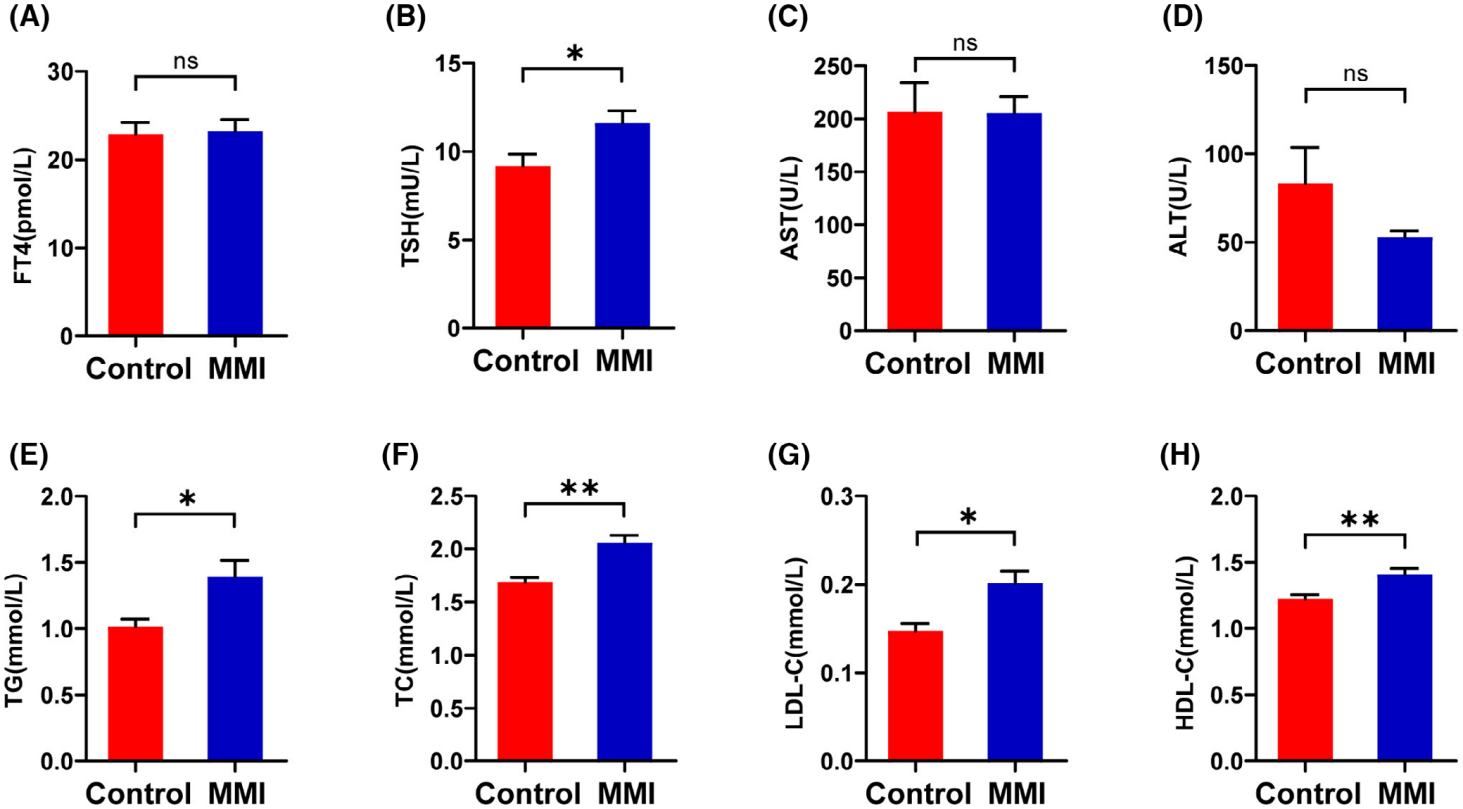
Serum biochemical indices in mice following a 12‐week chemical induction with methimazole (MMI). (A and B) Thyroid function parameters (serum FT4 and TSH levels). (C and D) Liver function indices (serum AST and ALT levels). (E–H) Lipid profiles (serum TG, TC, LDL‐C, HDL‐C levels). Data were presented as mean ± SEM (*n* = 7). ns, not statistically significant; *p < .05; **p < .01.

Subclinical hypothyroidism was induced chemically by MMI. As expected, after 12 weeks of MMI administration, the MMI and control groups did not differ significantly in serum FT4 levels, whereas the MMI group was markedly increased in serum TSH level (*p* < .05), indicating subclinical hypothyroidism (Figure 1A,B). The relevant data shown in Table 1 partially reflect that the extent of injury in thyroid function within the MMI group is consistent. Notably, the MMI group had higher lipid levels in comparison with the control group (Figure 1E–H). Furthermore, considering that long‐term MMI administration may cause liver injury in mice, the liver function indices in the two groups were evaluated. The results indicated that there were no significant differences in serum AST or ALT levels between the two groups (Figure 1C,D). Thus, the SCH mouse model was successfully and safely conducted by a chemical‐induced method and accompanied by dyslipidemia.

**TABLE 1:** The serum FT4 and TSH levels of the control and MMI groups.

	Control group (*n* = 7)	MMI group (*n* = 7)	*p*‐value
FT4 (pmol/L)	22.86 ± 3.52 (19.06–27.04)	23.17 ± 3.67 (17.51–28.63)	.881
TSH (mU/L)	9.18 ± 1.77 (6.65–11.30)	11.60 ± 1.84 (8.93–13.75)	.**027**

*Note*: The data were presented as means ± standard deviation (minimum‐maximum). The bolded value indicates that the p‐value is less than 0.05.

### 3.2 Gut microbiota composition in SCH mice

**FIGURE 2:**
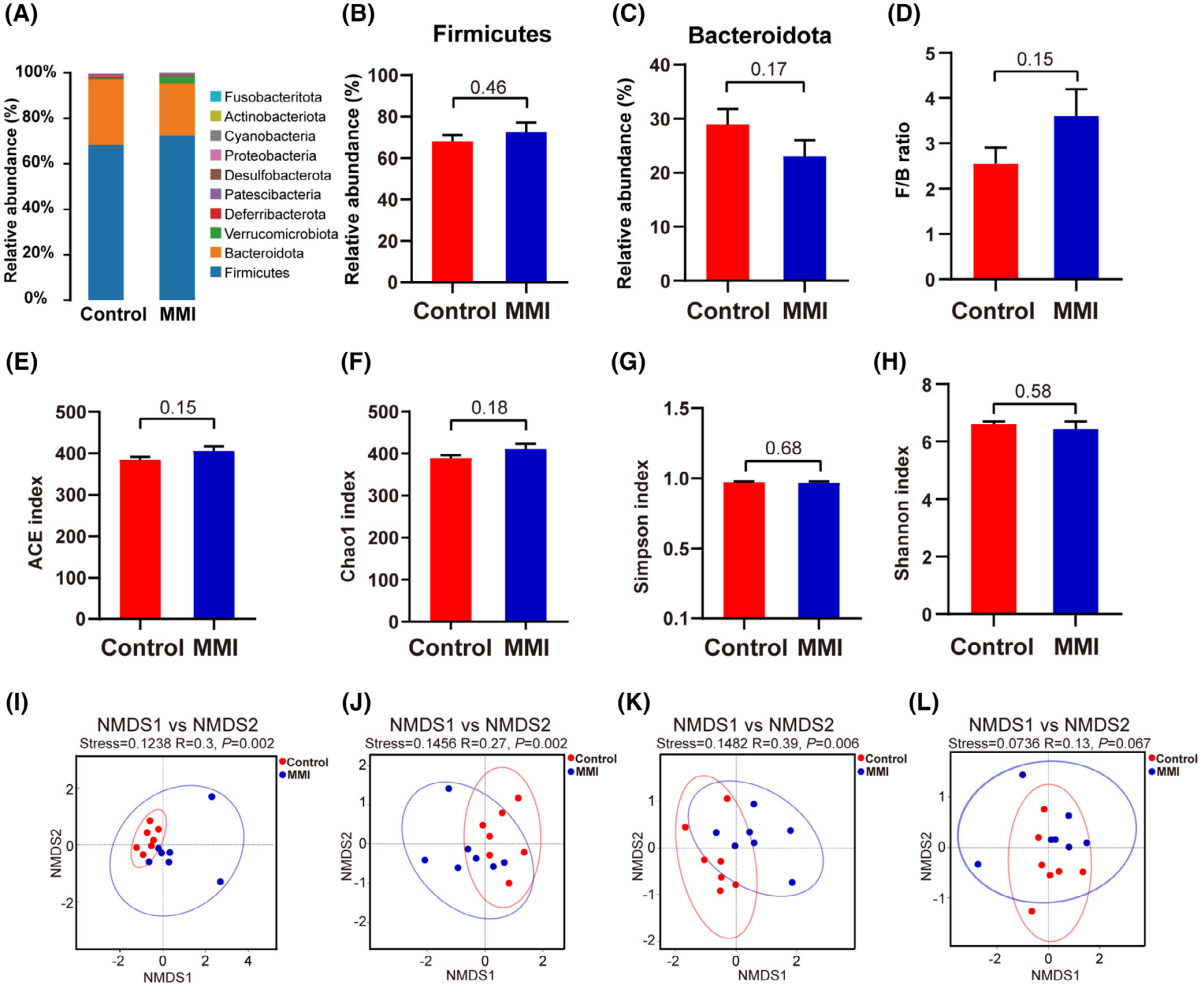
Taxonomic annotation and the diversity analysis of gut microbiota from MMI and control groups (*n* = 7). (A) Phylum‐level relative abundance of intestinal flora. Two dominant phyla were highlighted in red rectangles. (B–D) The relative abundance of two dominant phyla, including *Firmicutes* (B) and *Bacteroidota* (C), and F/B ratio (D). (E–H) A comparison of the α‐diversity indices (Chao1, ACE, Simpson, and Shannon) among two groups of gut microbes. (I–L) The NMDS analysis based on binary Jaccard distance (I), unweighted Unifrac distance (J), Bray‐Curtis distance (K), and weighted Unifrac distance (L).

Gut microbiota composition was analyzed using fecal samples from mice in two groups. OTU cluster analysis identified 555 OTUs across all samples, with 486 of them being shared by the two groups and 39 in the MMI group being unique (Figure S1). A rarefaction curve confirmed that the sequencing data were adequate to reflect the species diversity and richness in the samples (Figure S2). Based on taxonomic annotation, *Firmicutes* and *Bacteroidota* emerged as the dominant phyla (Figure 2A and Figure S3). Interestingly, neither their relative abundances nor the relative abundance ratio of *Firmicutes* and *Bacteroidota* (F/B ratio) differed statistically between groups (Figure 2B–D). α‐diversity shows species abundance and diversity in the gut microbial community of samples. As shown in Figure 2E,F, ACE index and Chao 1 index, which all reflect species richness, indicated increasing trends in SCH mice relative to control mice, though these differences did not reach statistical significance. Simpson index and Shannon index, reflecting species diversity, also did not observe any marked differences between the groups (Figure 2G,H). β‐diversity of gut microbiota between the two groups was conducted using NMDS analysis based on the weighted and unweighted algorithms (Figure 2I–L). The stress values of all four algorithms were below 0.2, which signified that the results of NMDS analysis were reliable. ANOSIM analysis indicated significant differences in β‐diversity between groups compared to within groups (Figure S4). Notably, an ANOSIM analysis using Bray‐Curtis distance produced the highest *R* value (*R* = .39), highlighting that gut microbiota β‐diversity differences between groups were influenced largely by species abundance, but the differences were not large enough. These findings indicated that subclinical hypothyroidism did not lead to noticeable alterations in the gut microbiota balance in mice.

### 3.3 Screening for differential microbiota between the two groups

**FIGURE 3:**
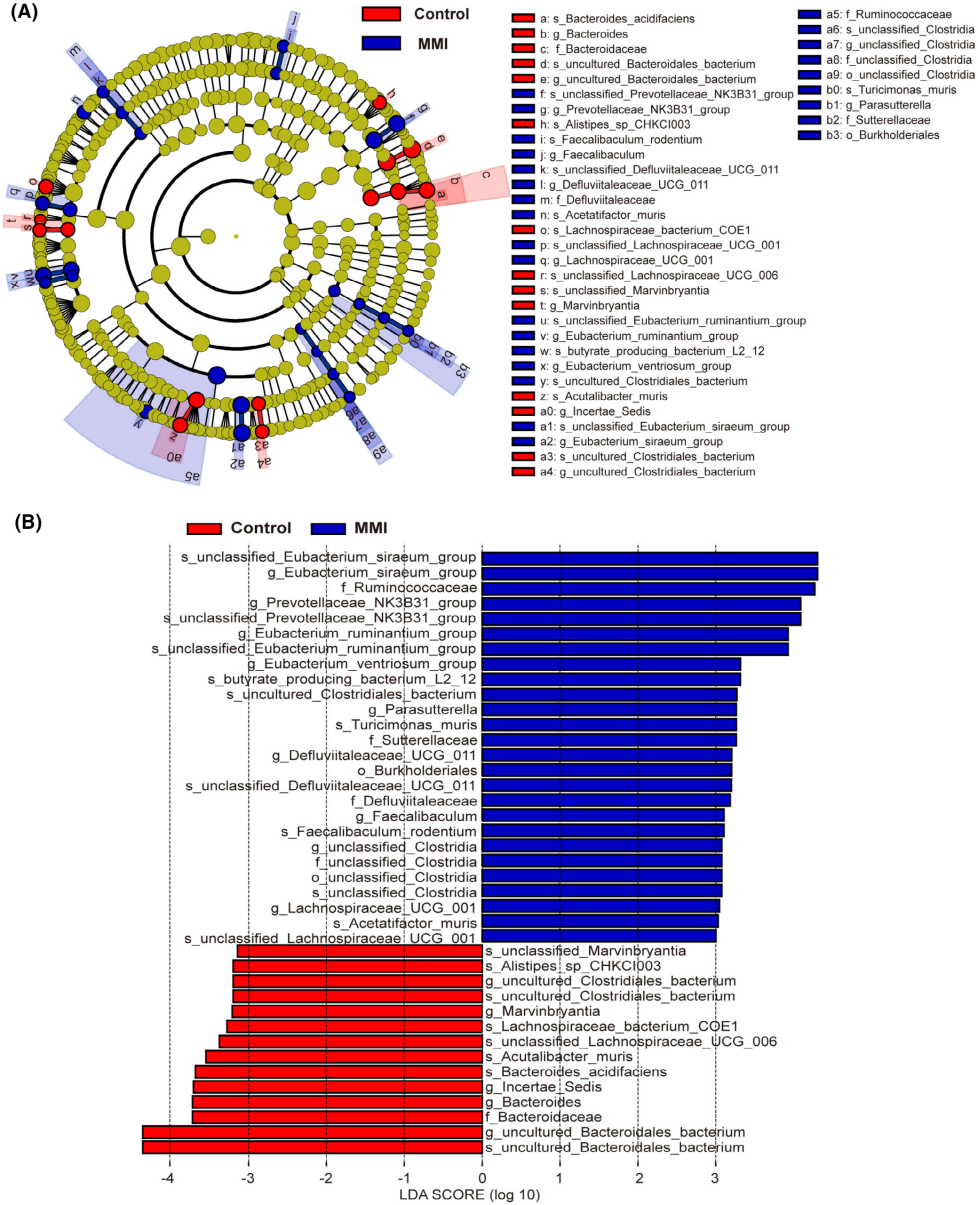
The differential microbiota at various taxonomic levels between two groups was determined by LEfSe analysis (LDA value >3, *p* < .05). (A) The evolutionary branch diagram. (B) The LDA value distribution histogram.

To further assess the effect of subclinical hypothyroidism on gut microbiota, differential microbiota among the groups at different taxonomic levels was determined by LEfSe analysis (Figure 3). Five differential families and 14 differential genera between the two groups were identified in this study. Specifically, at the family level, SCH mice exhibited enrichment in 4 families while the control mice had enrichment in *Bacteroidaceae*. At the genus level, 9 genera including *Eubacterium_siraeum*, *Prevotellaceae‐NK3B31*, *Parasutterella*, *Faecalibaculum*, and *Eubacterium_ruminantium* had higher abundances in the SCH mice, whereas 5 genera, including *Bacteroides*, had lower abundances compared with the control group.

### 3.4 Correlations between gut microbiota composition and biochemical indices in mice

**FIGURE 4:**
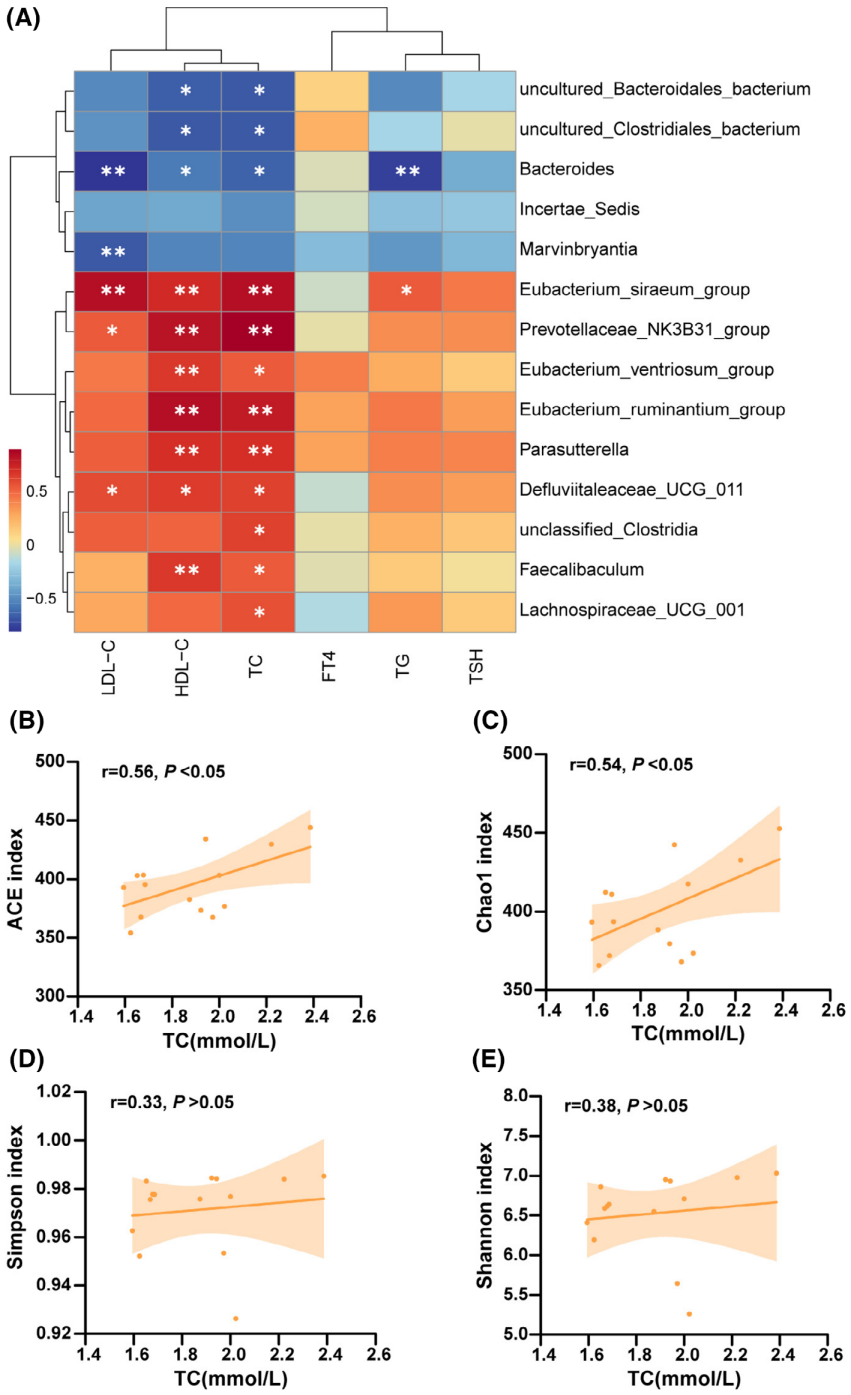
The Spearman correlation analysis between gut microbiota and biochemical indices. (A) The heatmap showed the correlation between 14 differential genera and serum biochemical indices (including thyroid function indices and lipid profiles). (B–E) An analysis of the correlation between TC level in serum and gut microbiota α‐diversity indices. The correlation coefficient *r* > .3 for the small cell with the significance mark (* or **) in the heatmap. *p < .05; **p < .01.

In the next step, we explored the correlations between differential genera identified by LEfSe analysis and the indices of thyroid function and lipid profiles in serum (Figure 4). We found that the relative abundances of differential genera showed no significant correlation with FT4 and TSH levels (Figure 4A, all *p* > .05). Interestingly, serum lipid profiles exhibited markedly significant correlation with the relative abundance of multiple differential genera. Specifically, *Eubacterium_siraeum* and *Prevotellaceae_NK3B31* were positively related to serum TC, TG, LDL‐C, and HDL‐C levels. Also, *Eubacterium_ruminantium, Parasutterella*, and Faecalibaculum showed significant positive correlations with TC and HDL‐C levels in serum. However, *Bacteroides* was observed to be negatively associated with lipid profiles. Moreover, the ACE index and Chao1 index, which both reflect species richness, showed significant positive correlations with serum TC levels, but not the Simpson index and Shannon index, which reflect species diversity (Figure 4B–E). Nevertheless, we did not observe that thyroid function indices (FT4 and TSH) and other serum lipid indices (TG, LDL‐C, and HDL‐C) were markedly related to α‐diversity indices of gut microbiota (data not shown). Based on these findings, the alteration in gut microbiota abundance may be closely linked to dyslipidemia associated with subclinical hypothyroidism.

### 3.5 The effect of intestinal microbiota imbalances on thyroid function in mice

**FIGURE 5:**
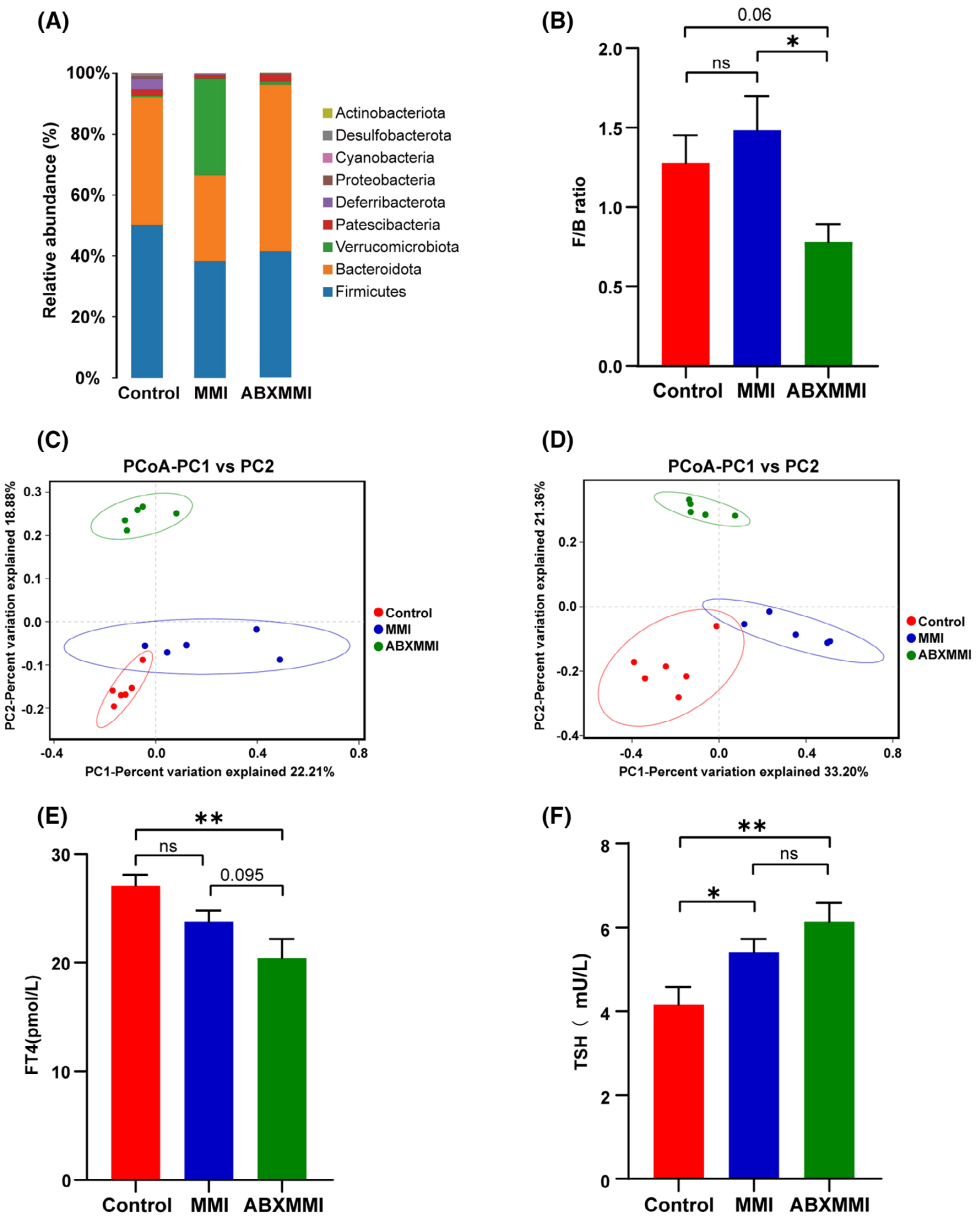
The composition of gut microbiota and thyroid function indices in three groups (*n* = 5–6). (A) Phylum‐level relative abundance of intestinal flora (the detailed information about each phylum in the three groups is shown in Table S1). (B) F/B ratio. (C and D) PCoA analysis based on binary Jaccard distance (C) and Bray‐Curtis distance (D). (E and F) FT4 (E) and TSH (F) levels. ns, not statistically significant; *p < .05; **p < .01.

For investigating  futher the relationship between thyroid function and gut microbiota, Polymyxin B and neomycin, non‐absorbable antibiotics known to deplete gut bacteria, were given to mice.^28^ As expected, antibiotic administration significantly disrupted the gut microbiota balance of mice (Figure 5A–D and Table S1). The F/B ratio is commonly used as one of the indexes for reflecting intestinal microbiota homeostasis.^29^ Compared with the control group, the F/B ratio in the MMI combined with antibiotic‐disturbed (ABXMMI) group had a substantially decreased trend (*p* = .06) and that in the MMI group did not significantly change (Figure 5B). What's more, the analysis and visualization of β‐diversity using PCoA analysis revealed a clear difference between the ABXMMI group and the other two groups (Figure 5C,D). Unweighted pair group method with arithmetic mean (UPGMA) also showed similar results (Figure S5). Crucially, in comparison with the control group, decreased FT4 levels and increased TSH levels were observed in the ABXMMI group (all *p* < .05), manifesting overt hypothyroidism in these mice. Compared with the MMI group, which presented subclinical hypothyroidism, the serum FT4 level in the ABXMMI group exhibited a decreasing trend, and the serum TSH level exhibited an increasing trend, although these differences did not reach statistical significance. These findings suggested that intestinal microbiota imbalances exacerbated hypothyroidism, strongly proving that gut microbial dysbiosis can affect thyroid function.

## 4 DISCUSSION

Gut microbiota is a complex and diverse microbial community closely associated with various thyroid disorders. In our study, mice treated with MMI exhibited unchanged serum FT4 levels but had elevated serum TSH levels and lipid profiles, indicating subclinical hypothyroidism. Although significant changes in gut microbiota balance were not observed in SCH mice, differential genera between the two groups were closely linked to serum lipid levels. Notably, gut microbiota imbalance following antibiotics disruption exacerbated hypothyroidism, suggesting that gut microbiota plays an important role in regulating thyroid function, and its alteration may be related to the degree of thyroid dysfunction.

The F/B ratio characteristically reflects whether intestinal flora is unbalanced and is typically stable in the normal population but alters in patients with various endocrine disorders.^30^, ^31^, ^32^ Su et al. found a significantly higher F/B ratio in patients with primary hypothyroidism compared with the healthy controls.^33^ Nevertheless, the F/B ratio in the SCH mice only showed an increasing trend in our study (*p* > .05). This discrepancy might be attributed to the varying degrees of hypothyroidism. In addition, the α‐diversity analysis revealed that the ACE index and Chao 1 index in SCH mice exhibited increasing trends compared with normal mice, though without statistical significance, which is in line with the previous study in SCH rats after subtotal thyroidectomy.^20^ Interestingly, a previous study found that the ACE index and Chao 1 index in primary hypothyroidism patients elevated compared with normal controls.^33^ These results implied that subclinical hypothyroidism might first manifest as changes in gut microbiota species richness. The Simpson index and Shannon index were not markedly different between SCH and control mice, indicating that subclinical hypothyroidism has not yet significantly impacted gut microbiota species diversity. The NMDS and ANOSIM analyses confirmed that gut microbiota β‐diversity in SCH mice significantly differs compared with the control group, primarily due to differences in species richness. Nevertheless, considering that β‐diversity difference between the two groups was not sufficiently large (*R* = .39), we propose that subclinical hypothyroidism, characterized by mildly abnormal thyroid function, may not yet cause significant alterations in gut microbiota homeostasis but rather only affect the relative abundance of specific intestinal floras. Further longitudinal studies in mice or patients are needed to substantiate this conclusion.

In this study, nine genera enriched in the subclinical hypothyroidism group were identified by LEfSe analysis, including *Eubacterium_siraeum*, *Parasutterella*, *Eubacterium_ruminantium*, and *Faecalibaculum*. The abundance of *Eubacterium_siraeum* markedly elevated in patients with type I diabetes and severe irritable bowel symptoms, suggesting its increased abundance may be associated with gastrointestinal symptoms in endocrine disorders, including SCH.^34^, ^35^ Previous research found that *Parasutterella* abundance was elevated in patients with Hashimoto's thyroiditis with hypothyroidism.^36^ Previous research found that the abundance of *Parasutterella* and *Eubacterium_ruminantium* positively related to the severity of malaise and constipation in thyroid carcinoma patients after thyroidectomy and accompanied by hypothyroidism.^37^ *Faecalibaculum* abundance was found to be reduced in Hashimoto's thyroiditis and differentiated thyroid carcinoma patients, which is inconsistent with our findings.^16^, ^37^ However, Ye et al. observed that *Faecalibaculum* abundance was related to both weight gain and dyslipidemia in mice administered high‐fat diets, suggesting a possible link between *Faecalibaculum* and dyslipidemia due to subclinical hypothyroidism.^38^ In brief, these genera enriched in the SCH mice deserve attention and should be studied further.

A previous clinical study explored the relationship between subclinical hypothyroidism and dyslipidemia from the perspective of gut microbiota.^18^ The results did not find a significant correlation between dyslipidemia and gut microbiota in SCH patients. However, their study population, with a higher average age (64.0 ± 8.3 years), included patients who had been treated with L‐thyroxine and those with concomitant hypertension and diabetes. The lipid data of the study subjects might have been affected by drug interventions such as metformin or glipizide. To minimize the influence of other factors on dyslipidemia, we constructed the SCH mouse model and rigorously investigated the relationship between intestinal flora and dyslipidemia in subclinical hypothyroidism. Our correlation analysis revealed that differential genera abundances between the two groups did not significantly correlate with the serum FT4 and TSH levels in mice, which suggests that the thyroid function alteration in SCH is not a major factor influencing gut microbiota differences between the groups. Notably, there were close relationships between the abundances of differential genera and serum lipid levels. It has been reported that the alteration of lipid metabolism promotes the development of metabolic obesity, and *Eubacterium_siraeum* may be involved in the development of metabolically healthy obesity.^39^ Our study also revealed that *Eubacterium_siraeum* relative abundance was related positively to serum TC and TG levels, further verifying its relationship with lipid metabolism. Furthermore, a study about thyroidectomized patients with hypothyroidism also indicated that *Eubacterium_ruminantium* abundance was positively related to TC and LDL‐C levels in serum.^37^ A previous study showed that *Parasutterella* abundance was related to the activation of the fatty acid synthesis pathway and decreased during weight loss intervention, which coincides with our finding that *Parasutterella* abundance was positively associated with serum TC levels.^40^ *Faecalibaculum* abundance was related positively to serum TC, TG, and LDL‐C levels in obese mice; a similar positive correlation was found in SCH mice in our study.^38^ *Bacteroides* is a common genus of intestinal flora, and its abundance was observed to have a marked correlation with lipid levels in our study. A study demonstrated that body weight and lipid profile were reduced in hyperlipidemic rats after oral administration of *Bacteroides vulgatus*.^41^ The previous study showed that statin intolerance was more prevalent in hypothyroidism patients.^42^ A clinical study reported that the serum TSH level and lipid levels of pregnant women significantly reduced after treatment with probiotics and prebiotics, manifesting the feasibility of probiotics for treating SCH and dyslipidemia.^43^ Therefore, a probiotic complex containing *Bacteroides* may be a novel, safe, and effective lipid‐lowering treatment. In addition, our study found that serum TC levels had a significant positive correlation with indicators reflecting gut microbiota species richness, including the ACE index and Chao1 index. This suggests that gut microbiota abundance alteration may partly contribute to abnormal serum TC levels in subclinical hypothyroidism. Altogether, the gut microbiota species richness and certain flora abundance in subclinical hypothyroidism are closely associated with dyslipidemia. These findings provide new lights into improving dyslipidemia in SCH patients. We need to conduct more clinical and animal research in the future to validate our findings and explore the underlying mechanisms.

Gut microbiota participated in thyroid hormone homeostasis, including the enterohepatic cycle, iodothyronine metabolism, and absorption of oral thyroxine.^13^, ^44^ Vought et al. explored the influence of the alteration in the intestinal flora on iodine absorption by administering kanamycin, a non‐absorbable oral antibiotic, to rats.^45^ Compared with controls, rats treated with kanamycin for 42 and 72 days had a lower three‐hour thyroidal radioiodine uptake. DiStefano et al. demonstrated reversible bacterial binding of triiodothyronine and thyroxine in normal rats' feces and cecum contents, which reduced or even eliminated in rats treated with antibiotics, indicating that gut microbiota might serve as a reservoir for thyroid hormones.^46^ Additionally, Rutgers et al. found that the presence of intestinal flora in rats might facilitate reabsorption of triiodothyronine after hydrolysis.^47^ Researchers have detected type II iodothyronine 5′‐monodeiodinases within the human intestine, adding to the evidence supporting gut microbiota's involvement in thyroid hormone metabolism.^48^ Interestingly, a clinical study indicated that the L‐thyroxine dose to keep the levels of TSH in serum steady in patients with subclinical hypothyroidism was related to gut microbiota composition.^18^ Spaggiari et al. reported that the VSL#3® probiotic supplementation could reduce the frequency of L‐thyroxine dosage adjustments and prevent fluctuations in serum thyroid hormone levels.^49^ In our study, we used Polymyxin B and neomycin, non‐absorbable antibiotics, to deplete gut bacteria. The results showed no statistically significant differences in serum FT4 and TSH levels between the ABXMMI and MMI groups. This suggests that gut microbiota imbalance alone may not significantly alter thyroid function within 12 weeks. Similarly, Moshkelgosha et al. found that the administration of vancomycin alone in mice, which is not readily absorbed, disturbed the gut microbiota homeostasis but did not significantly change the serum level of total thyroxine.^50^ However, compared with the MMI group, the ABXMMI group showed a decreasing trend in the serum FT4 level and an increasing trend in the serum TSH level, leading to overt hypothyroidism in the ABXMMI mice and indicating a more severe condition than subclinical hypothyroidism observed in MMI‐treated mice. These findings showed that gut microbiota imbalance exacerbated hypothyroidism in MMI‐induced mice, providing further direct support for the idea that gut microbiota regulates thyroid hormone homeostasis.

In conclusion, this study could help further understand the relationship between gut microbiota and subclinical hypothyroidism, and provide new insights for the adjuvant management of this disease. Our findings indicated that subclinical hypothyroidism, characterized by mildly abnormal thyroid function, had not yet significantly altered gut microbiota homeostasis, but the imbalance of gut microbiota aggravated hypothyroidism. These results manifest that gut microbiota plays an essential role in the development of the thyroid dysfunction disease. Moreover, our research uncovered an association between gut microbiota and dyslipidemia in SCH, pointing us toward new avenues for the mechanism research of dyslipidemia in subclinical hypothyroidism. However, this study is still exploratory research. More animal studies and large‐sample, multicenter clinical trials are required to validate these findings in the future. Furthermore, the role of gut microbiota on thyroid function warrants further investigation. In the future, we will conduct more experiments, such as antibiotic withdrawal experiments and appropriate probiotics supplementation, to dig deeper into this project.

## AUTHOR CONTRIBUTIONS

Ru Wang, Xiaqing Yu, and Haidong Cai performed the research and wrote the manuscript. Ganghua Lu, Dingwei Gao, Mengyu Zhang, and Li Chai analyzed and interpreted the data. Zhongwei Lv and Wanwan Yi conceived the research and reviewed the manuscript.

## FUNDING INFORMATION

This study was sponsored by National Natural Science Foundation of China (Grant No. 82071964), National Natural Science Foundation of China for Young Scholars (Grant No. 82202203), Shanghai Leading Talent program sponsored by Shanghai Human Resources and Social Security Bureau (Grant No. 2019), Shanghai Municipal Health Commission (Grant No. GWVI‐11. 2‐YQ51), Shanghai Municipal Commission of Economy and Informatization (Grant No. 23SHS06500‐04), and Shanghai Tenth People's Hospital (Grant No. 2021SYPDRC065, 2023YJXYSB011).

## DISCLOSURES

All the authors have no conflicts of interest to disclose.

## Supporting information


Data S1.

## Data Availability

The data that support the findings of this study are available in the Materials and Methods, Results, and/or Supplemental Material of this article.
