# Supplementary material for: Gut microbiota alteration was related to subclinical hypothyroidism and dyslipidemia in mice
Source: FASEB J. 2025 Mar 7;39(5):e70445. doi: 10.1096/fj.202402289RR (PMC11887603; doi:10.1096/fj.202402289RR)
Supplement: Supplementary file 1 — Data S1. [file FSB2-39-e70445-s001.docx]

**Gut Microbiota Alteration Was Related to Subclinical Hypothyroidism and Dyslipidemia in Mice**

Ru Wang^1, #^, Xiaqing Yu^1, #^, Haidong Cai^1, #^, Ganghua Lu^1^, Dingwei Gao^1^, Mengyu Zhang^1^, Li Chai ^1^, Wanwan Yi^1,^ ^*^, Zhongwei Lv^1,2, *^

^1^ Clinical Nuclear Medicine Center, Imaging Clinical Medical Center, Institute of Nuclear Medicine, Institute of Clinical Mass Spectrometry Applied Research Center, Department of Nuclear Medicine, Shanghai Tenth People's Hospital, School of Medicine, Tongji University, Shanghai 200072, China.

^2^ Shanghai Public Health Clinical Center, Fudan University, Shanghai 200003, China.

^#^ Ru Wang, Xiaqing Yu and Haidong Cai should be considered joint first authors.

^*^These authors contributed equally to this work.

^*^Correspondence:

Zhongwei Lv (Email: [Lvzwjs2020@163.com](mailto:Lvzwjs2020@163.com));

Wanwan Yi (Email: 1911608@tongji.edu.cn).

**Supplemental Material**

**
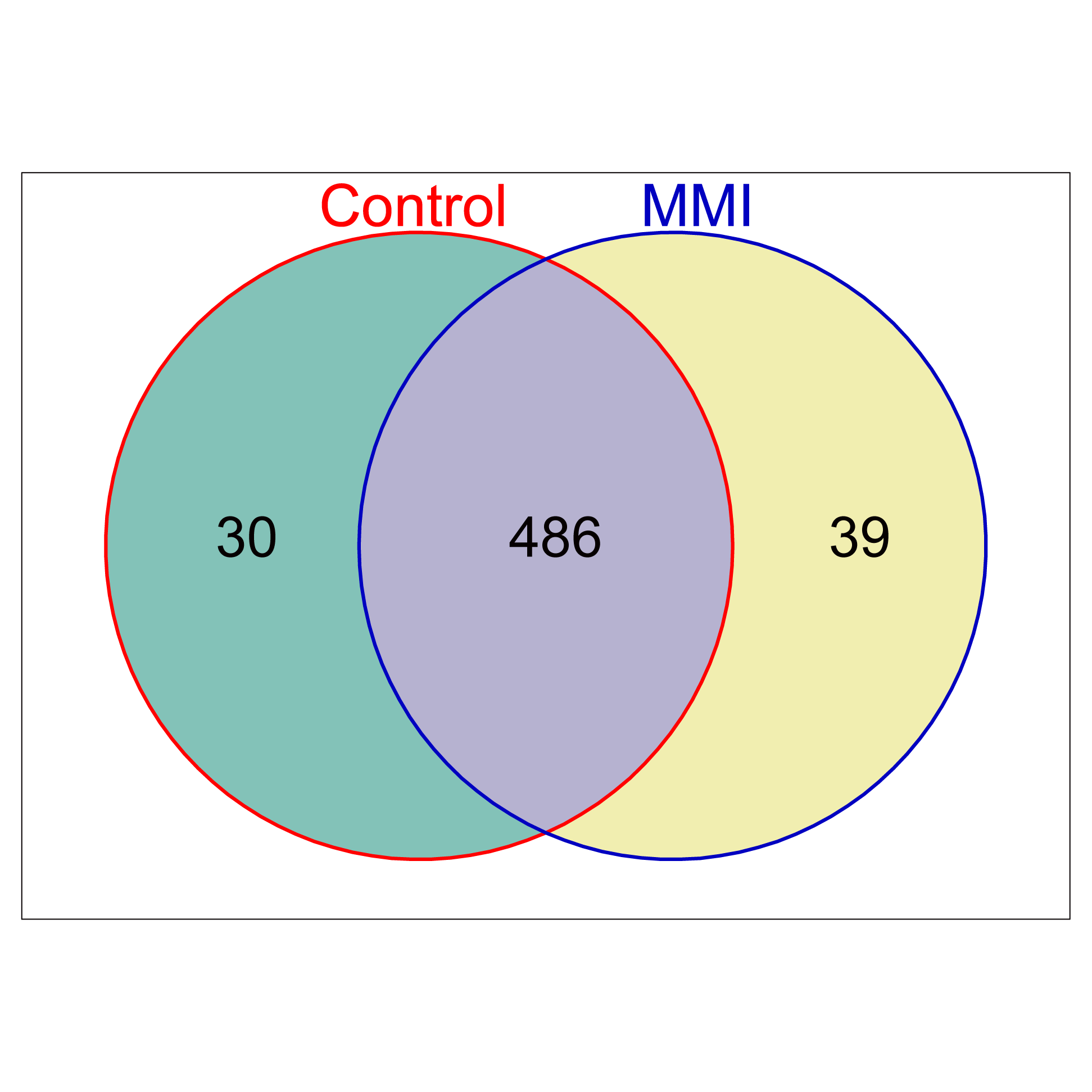
**

FIGURE S1 The Venn diagram based on the OTU cluster analysis of gut microbiota in two-group mice.

**
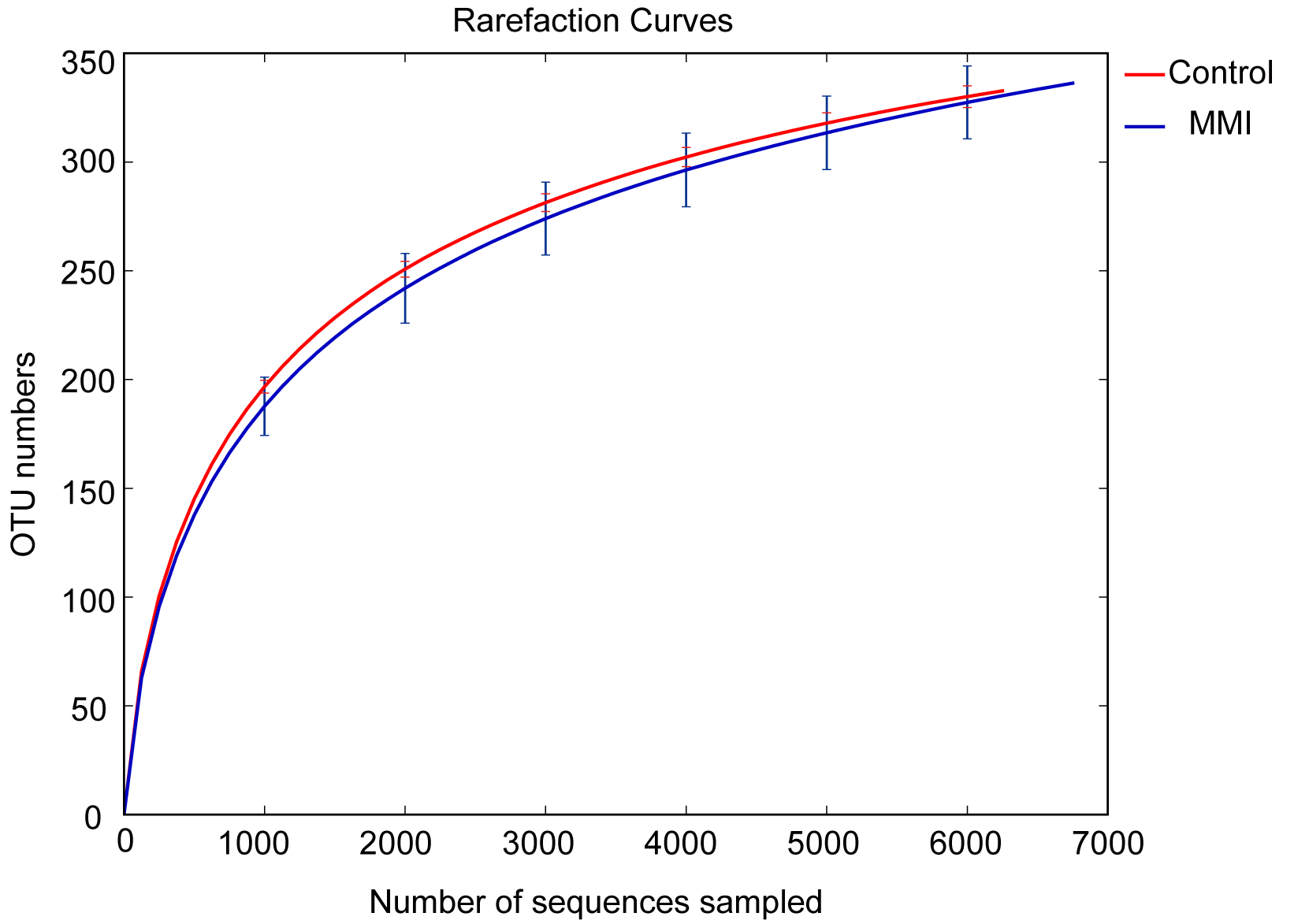
**

FIGURE S2 The rarefaction curves base on the extracted sequencing data and the corresponding OTUs.


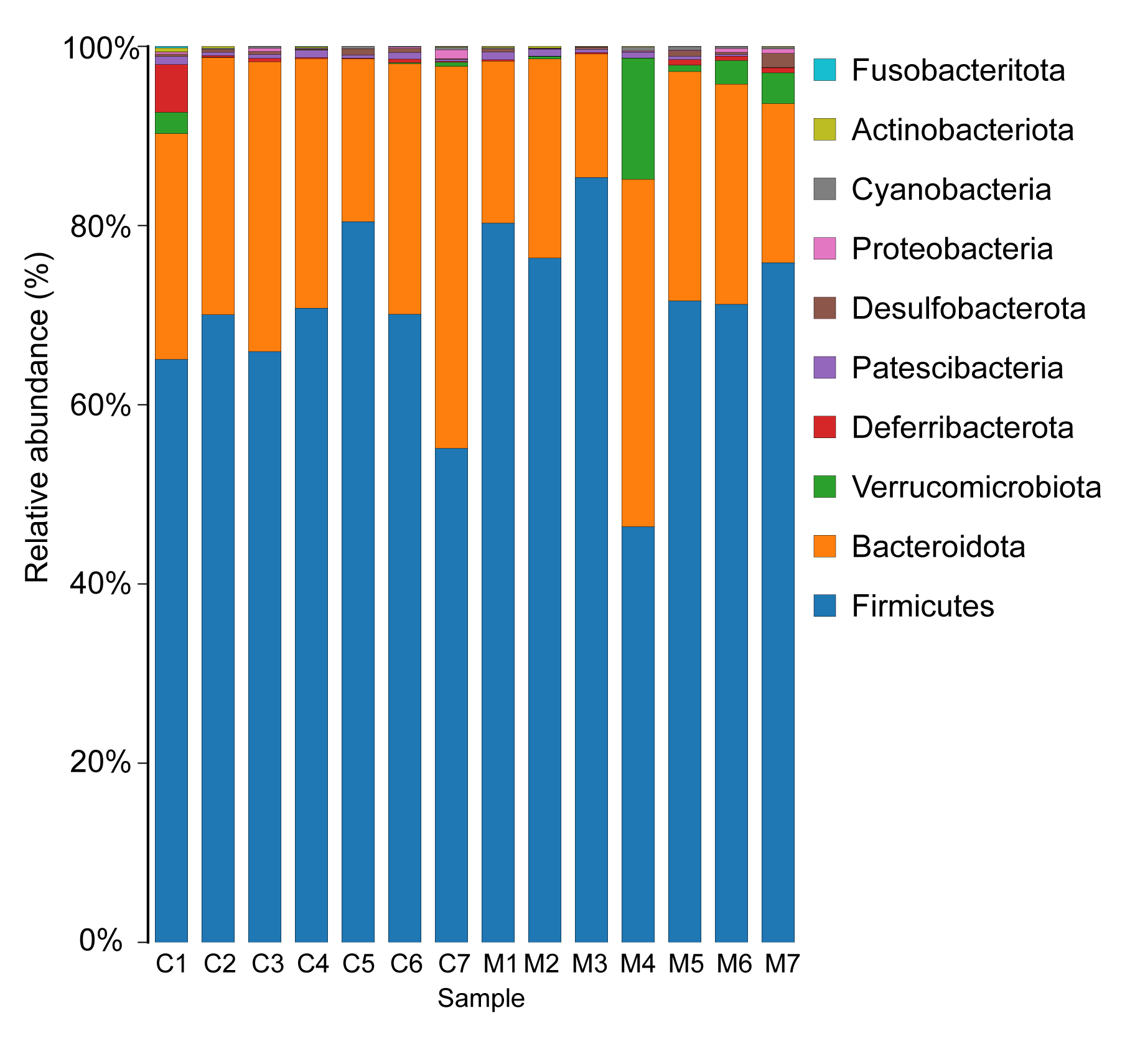


FIGURE S3 The relative abundance of intestinal flora in each sample in control (C1-C7) and SCH mice (M1-M7) at the phylum level.


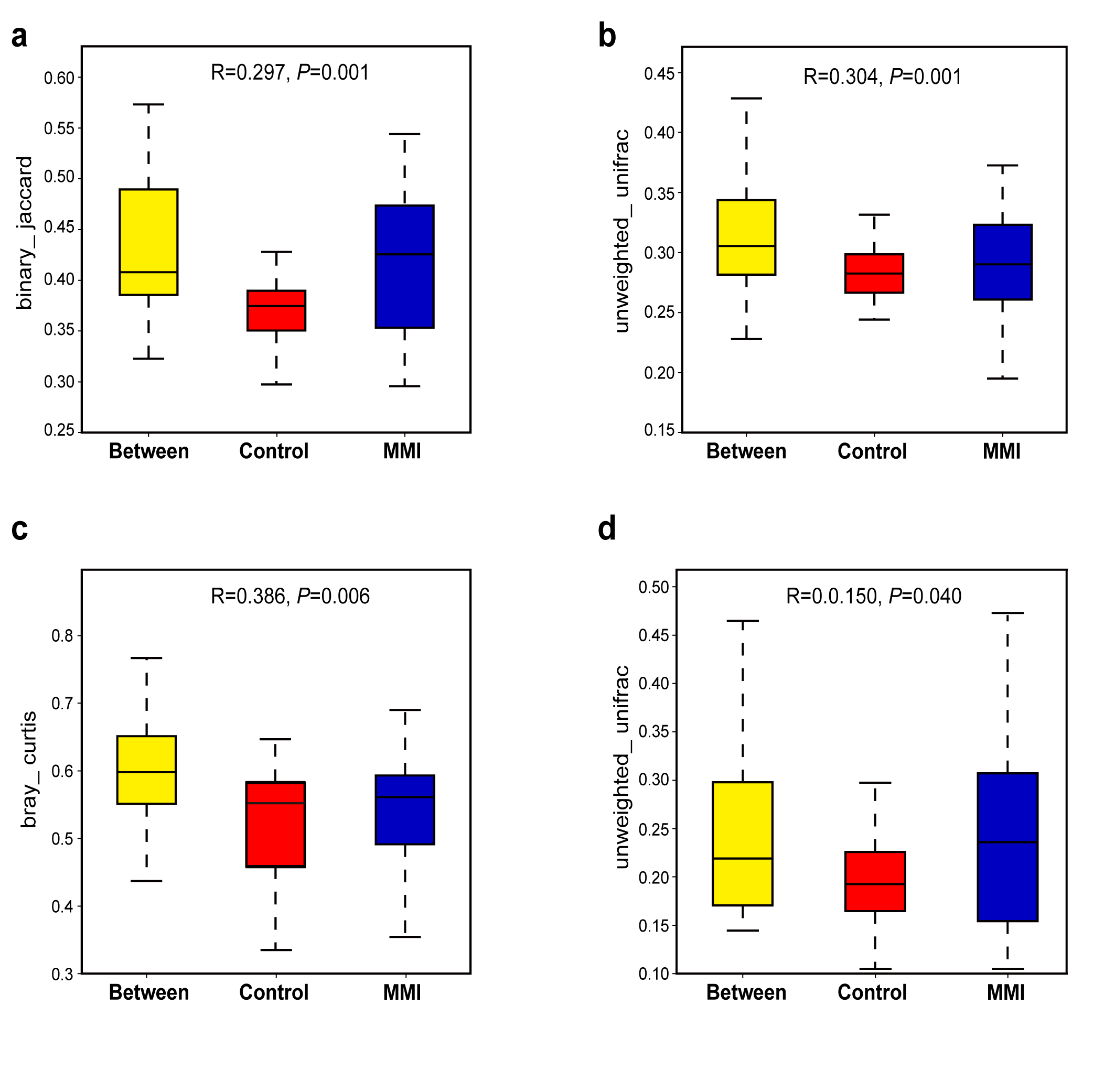


FIGURE S4 The ANOSIM analysis. a. based on binary Jaccard distance. b. unweighted Unifrac distance, c. Bray-Curtis distance, d. weighted Unifrac distance.

TABLE S1 The proportion of each phylum in three groups

| Phylum | Control group (%) | MMI group (%) | ABXMMI group (%) |
| --- | --- | --- | --- |
| Actinobacteriota | 0.03 | 0.02 | 0.03 |
| Desulfobacterota | 0.41 | 0.15 | 0 |
| Cyanobacteria | 0.44 | 0.22 | 0.25 |
| Proteobacteria | 0.94 | 0.22 | 0.36 |
| Deferribacterota | 3.43 | 0.21 | 0 |
| Patescibacteria | 1.92 | 0.88 | 2.1 |
| Verrucomicrobiota | 0.77 | 31.79 | 1.24 |
| Bacteroidota | 41.86 | 28.18 | 54.64 |
| Firmicutes | 50.19 | 38.33 | 41.38 |


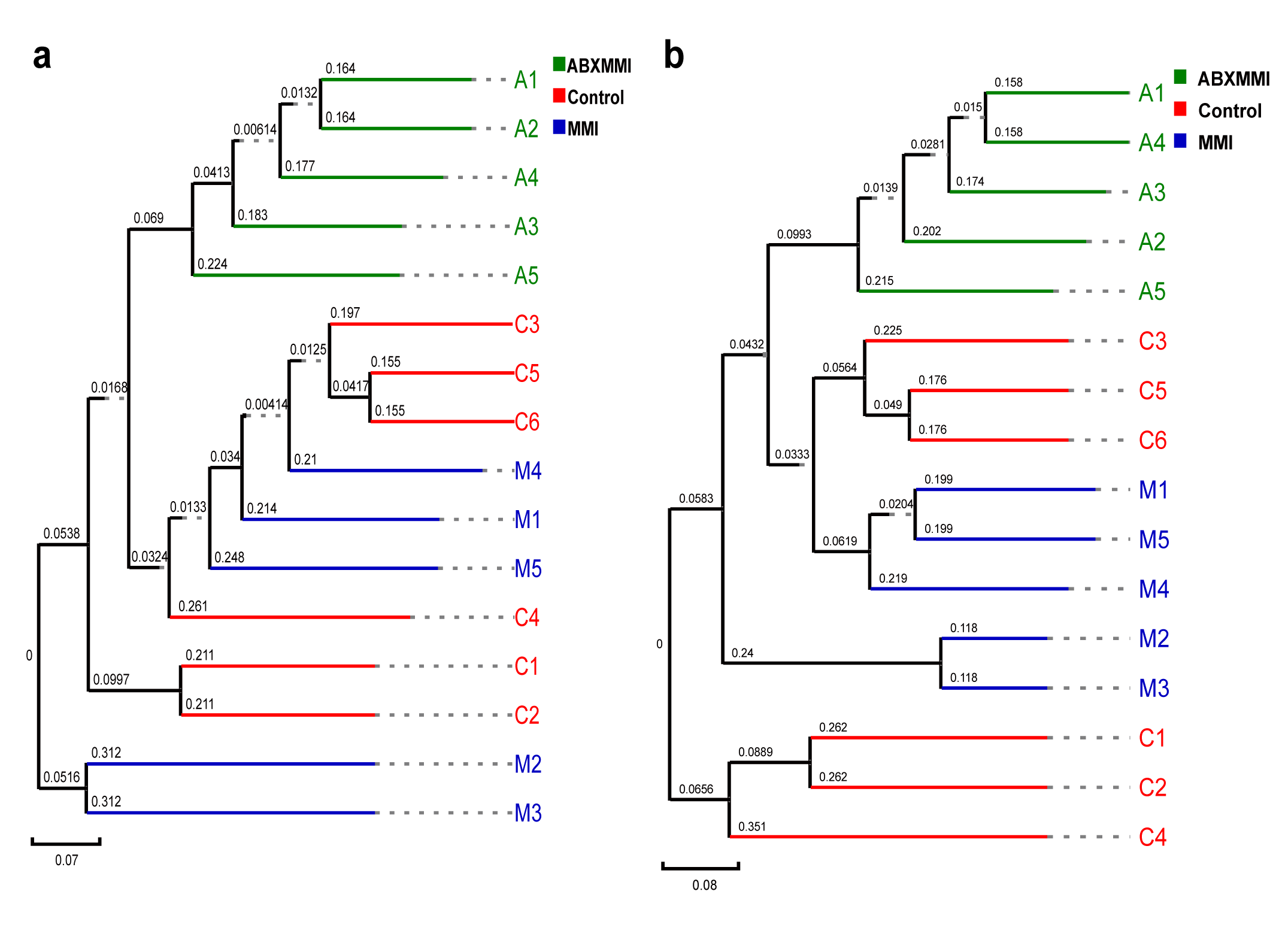


FIGURE S5 The Unweighted pair-group method with arithmetic means (UPGMA) hierarchical clustering analysis. a. based on binary Jaccard distance. b. based on Bray-Curtis distance. Green (A1-A5) represents the samples of MMI combined with antibiotic-disturbed group, red (C1-C6) represents the samples of control group, and blue (M1-M5) represents the samples of MMI group.
